# Peroneus longus intra-tendinous ganglion cyst

**DOI:** 10.1093/jscr/rjac279

**Published:** 2022-06-10

**Authors:** Yousef Tawfik Khoja, Abdulaziz Fahad Altammami, Mohammed Hashem Alattas

**Affiliations:** Department of Surgery, College of Medicine, Al-Imam Mohammad Ibn Saud Islamic University (IMSIU), Riyadh, Saudi Arabia; Department of Surgery, College of Medicine, Al-Imam Mohammad Ibn Saud Islamic University (IMSIU), Riyadh, Saudi Arabia; Department of Orthopedic Surgery, Security Forces Hospital, Riyadh, Saudi Arabia

## Abstract

Ganglion cysts arising from tendons are uncommon lesions with an unknown cause. We present a case report of a 38-year-old female was diagnosed with an intra-tendinous ganglion cyst of the peroneus longus. She complained of right ankle swelling for 1 year and associated with pain. MRI revealed a peroneus longus intra-tendinous ganglion below the lateral malleolus with a thin wall. The ganglion cyst was surgically excised while the structure of the peroneus longus tendon was preserved. The clinical and functional outcomes were satisfactory after 1 year without recurrence.

## INTRODUCTION

Ganglion cysts are benign soft-tissue tumors found in the hand, wrist, ankle and foot [1]. Although the cause of these lesions is unknown, a history of acute or recurrent chronic injury such as compression of the tendon against a bony prominence could explain the formation of cystic space [2]. The ganglion’s position relative to the muscle varies. However, intra-tendinous ganglion cysts are rare, especially in the peroneal tendon [3].

Ganglions arising from the Peroneus Longus tendon are uncommon. Few case reports of ganglion cysts arising from the muscle belly were published. Both Graves and Marano *et al.* reported on a ganglion found in the muscle belly of Peroneus Longus [4, 5]. Kumar *et al.* have reported a recurrent finding of a similar presentation [6]. To our knowledge, this is the first case report of an intra-tendinous ganglion cyst arising from the Peroneus Longus tendon.

## CASE REPORT

A 38-year-old female, not known to have any medical illness, presented to orthopedic outpatient clinic complaining of right ankle swelling with pain for 1 year. It was increasing with time & prolonged standing or walking. The swelling affected her footwear, and she tried changing them without success. She denied any prior trauma or similar lesions. She tried NSAIDs and ankle stabilization exercises with no significant improvement. Local examination of the swelling showed 3*3-cm tender, rubbery & mobile mass distally to lateral malleolus in her right ankle. It was not reducible or pulsatile. There was no sign of infection, and her distal neurovascular examination was normal.

**Figure 1:**
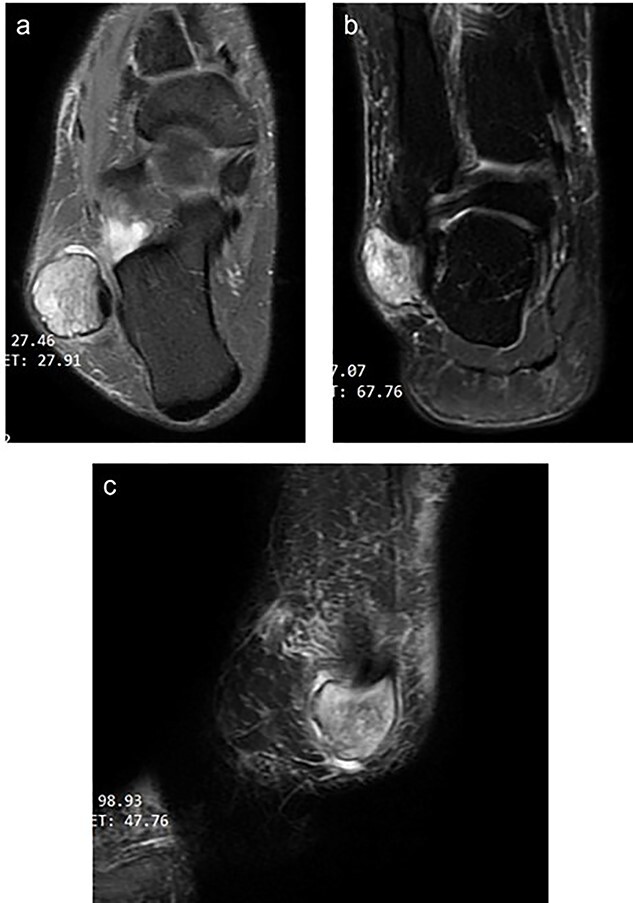
Magnetic resonance image of the right ankle (**a**: T2-weighted axial view **b**: T2-weighted coronal view **c**: fat-saturated T2 weighted sagittal view) showing a thin-walled well-circumscribed lesion within the peroneus longus tendon.

An AP, lateral & oblique X-ray views of her ankle were unremarkable for any boney or articular abnormalities. MRI with intravenous contrast showed a peroneus longus intra-tendinous ganglion cyst that measures 25*20 mm below the lateral malleolus with a thin wall (Fig. 1). The cyst contains fluid and fine septation within—a fine marginal mass enhancement after contrast uptake. The tibialis anterior and the Peronei tendons sheath showed tenosynovitis.

The patient was informed of her diagnosis and prognosis. To improve her condition, she required surgical excision of the cyst, which included surgical steps and if the lesion could be removed without affecting the integrity of the tendon or if it required reconstruction. She was also told about recurrence and peroneus tendon tear. She agreed to the surgery, and informed consent was obtained.

**Figure 2:**
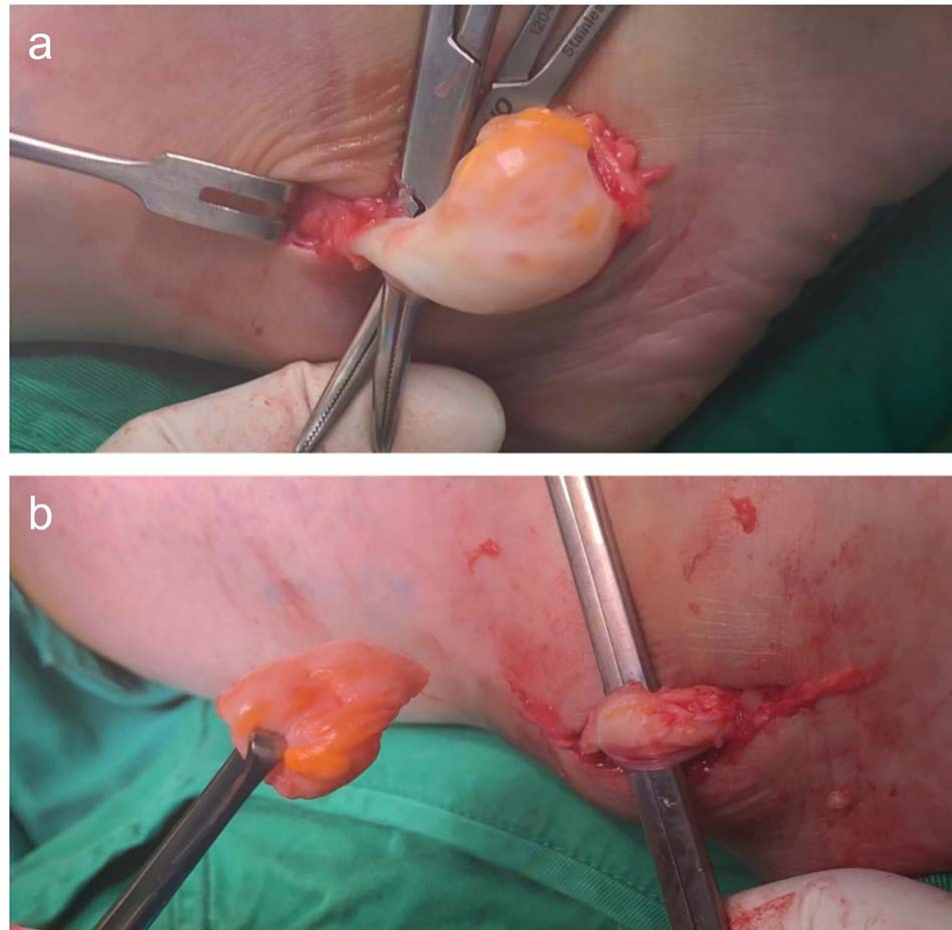
(**a**) Intra-tendinous ganglion cyst of the peroneus longus. (**b**) Ganglion cyst dissected free from surrounding tissues showing preserved peroneus longus tendon.

She was assessed by our anesthesia team and admitted to the hospital. In the operating room, she received general anesthesia and antibiotic. She was in a supine position with a tourniquet applied on her right thigh and inflated to 270 mmHg. She was prepped and draped in the usual sterile manner. A 3-cm incision was distal to lateral malleolus over the course of the peroneus tendon and the swelling. A cystic mass measuring 25*20 cm from the peroneal tendon was seen (Fig. 2a). After the isolation of the tendon, we were able to excise the cyst, and the defect was left without any suturing as the integrity tendon was normal (Fig. 2b). The excised mass was sent for histopathology. The wound was closed in the usual fashion then pressure dressing was applied. Tourniquet time was 56 min. She was extubated, and her postoperative recovery in the hospital was uneventful. The histopathology result of the cyst showed a band of collagenous fibrous tissue having congested vessels and entailing cystic spaces devoid of the epithelial and synovial lining. Areas of myxoid degeneration and lairy mucoid material are noted, consisting of a ganglion cyst.

She was instructed to mobilize as a non-weight for 2 weeks, then progressive weight bearing as tolerated for 4 weeks without any cast or support. She started ankle stabilization and proprioception exercises 6 weeks postoperatively. She was able to return to full activities without compromising her ankle eversion or plantarflexion and wear regular footwear. At 1 year follow-up, she did not show any sign of recurrence of the cyst.

## DISCUSSION

They can present within muscles, menisci and tendons [5]. Ganglion cysts arise from the peroneus longus tendon, often present with swelling over the lateral aspect of the leg that may cause pain, paraesthesia and weakness in the dorsiflexion of the foot; the neurological manifestations are due to the compression of the common peroneal nerve [7]. The diagnosis of a ganglion cyst starts with high clinical suspicion after examining the patient. Initially, a plain film study can be ordered to show a nonspecific soft-tissue density without any adjacent bony involvement [8]. The diagnosis can be made by magnetic resonance imaging, the preferred imaging method in such cases. Ganglion cysts are shown as homogeneous, low-intensity lesions on T1-weighted images with markedly increased signals on T2-weighted images [9].

Ganglion cysts can be resolved spontaneously at 52–58% without treatment [10]. Surgical excision is the preferred treatment for ganglion cysts [8]. The recurrence rate of lower extremity ganglion cysts after surgical excision is ~10% of cases [11]; hence, removing most of the surrounding degenerative capsular or tendon sheath collagen tissue is essential to help prevent recurrence [12]. Alternative therapeutic options include aspiration or injection of sclerosing agents and radiotherapy [13].

In 1956 Graves reported a ganglion cyst that occupied the muscle belly of the peroneus longus. Graves only mentioned X-ray examination as a preoperative assessment of the mass that showed ‘no involvement of the bone’. Fibrosarcoma or Rhabdomyosarcoma was thought to be a preoperative diagnosis due to limited diagnostic methods [4]. Kumar *et al.* described a recurrent peroneus longus ganglion that did not affect the neurological structure of the affected limb. Initially, the ganglion was treated by incision and drainage of the cyst. A month later, the lesion recurred. The ganglion was removed entirely with no cyst recurrence [6]. In the current report, anatomic preservation of the tendon was accomplished while managing to excise the entire ganglion cyst, which resulted in complete pain relief without recurrence and return to full functional activities.

## CONSENT

The patient has provided written consent for the publishing of the report.

## CONFLICT OF INTEREST STATEMENT

The authors declare no conflict of interest regarding the publication of this article.

## FUNDING

The authors declare that the research was conducted independently of any commercial or financial relationships.
